# A Novel Telehealth Exercise Program Designed for Rural Survivors of Cancer With Cancer-Related Fatigue: Single-Arm Feasibility Trial

**DOI:** 10.2196/59478

**Published:** 2025-01-10

**Authors:** Ryan J Marker, Andrew J Kittelson, Jared J Scorsone, Ian A Moran, John C Quindry, Heather J Leach

**Affiliations:** 1 Department of Physical Medicine and Rehabilitation University of Colorado Anschutz Medical Campus Aurora, CO United States; 2 School of Physical Therapy and Rehabilitation Science University of Montana Missoula, MT United States; 3 School of Integrative Physiology and Athletic Training University of Montana Missoula, MT United States; 4 Anschutz Health and Wellness Center University of Colorado Anschutz Medical Campus Aurora, CO United States; 5 Department of Health and Exercise Science Colorado State University Fort Collins, CO United States

**Keywords:** cancer-related fatigue, telehealth, physical activity, survivorship, digital health, lifestyle intervention, videoconference, symptom burden, symptom monitoring, geographic disparities, mHealth

## Abstract

**Background:**

Exercise interventions are among the best-known interventions for cancer-related fatigue (CRF). Rural survivors of cancer, however, report specific barriers to engaging in exercise programs and lack overall access to effective programs.

**Objective:**

The purpose of this investigation was to assess the feasibility of a novel telehealth exercise program designed specifically for rural survivors of cancer with CRF.

**Methods:**

A single-arm clinical trial of the BfitBwell Telehealth Program was performed. Based on an established clinical program, this adapted 12-week program addressed barriers previously reported by rural survivors by providing synchronous videoconference exercise sessions (2 per program), asynchronous exercise sessions using a personal training smartphone or internet app (3-5 per week), and regular symptom (CRF) monitoring using automated emailed surveys (every 2 weeks). Personalized exercise prescriptions containing aerobic and resistance activities were implemented by cancer exercise specialists. Symptom-triggered synchronous sessions were initiated for participants failing to improve in CRF, as identified by a reference chart of CRF improvements observed during a supervised exercise program. Eligible participants were adult survivors of any cancer diagnosis who had completed treatment with curative intent in the past 12 months or had no planned changes in treatment for the duration of the study, lived in a rural area, and were currently experiencing CRF. Feasibility was assessed by objective measures of recruitment, data collection, intervention acceptability and suitability, and preliminary evaluations of participant responses. CRF was the primary clinical outcome (assessed using the Functional Assessment of Chronic Illness Therapy—Fatigue Scale [FACIT-Fatigue]) and was measured before, after, and 6 months after program completion.

**Results:**

In total, 19 participants enrolled in the study, 16 initiated the exercise program, and 15 completed the program. A total of 14 participants were recruited through internet advertisements, and the total recruitment rate peaked at 5 participants per month. Participants completed 100% of initial and final assessments (30 assessments across all participants) and 93% (70/75 possible surveys across all participants) of emailed surveys and attended 97% (29/30 possible sessions across all participants) of synchronous exercise sessions. In total, 6 participants initiated symptom-triggered sessions, with 6 of 7 initiated sessions attended. The mean FACIT-Fatigue scores significantly improved (*P*=.001) by 11.2 (SD 6.8) points following the completion of the program. A total of 13 participants demonstrated at least a minimal clinically important difference in FACIT-Fatigue scores (≥ +3 points) at this time. FACIT-Fatigue scores did not significantly change from program completion to 6-month follow-up (n=13; mean change –1.1, SD 3.4 points; *P*=.29).

**Conclusions:**

Results from this investigation support the feasibility of the BfitBwell Telehealth Program and a subsequent efficacy trial. Novel program components also provide potential models for improving exercise program efficacy and efficiency through asynchronous exercise prescription and symptom monitoring.

**Trial Registration:**

ClinicalTrials.gov NCT04533165; https://clinicaltrials.gov/study/NCT04533165

## Introduction

Cancer-related fatigue (CRF) is one of the most common and functionally limiting symptoms reported by survivors of cancer, with an estimated prevalence of 49% to 62% [1-4]. It is present in over a quarter of survivors up to 10 years after the completion of treatment [5], and survivors regularly report CRF as the symptom preventing them from living a “normal” life as well as the cause of major life events such as employment changes [6]. Participation in exercise interventions is an established intervention for the improvement of CRF in survivors of cancer [7,8], and a multidisciplinary roundtable pronouncement by the American College of Sports Medicine (ACSM) indicated that there is strong evidence that exercise can significantly reduce CRF. The current ACSM recommendation for an effective exercise prescription to remediate CRF is moderate-intensity aerobic training at least 3 times per week for at least 30 minutes and moderate-intensity resistance training at least 2 times per week [9].

Survivors of cancer living in rural locations [10], however, commonly lack access to many supportive services compared to nonrural survivors, including clinical exercise programs [11]. Rural survivors commonly report many specific barriers to engaging in exercise programs including lack of knowledge of available programs, distance and transportation to programs, lack of access to a knowledgeable exercise provider, and lack of flexibility in programming (in terms of time and location) [12,13]. An overall lack of accessible exercise programs for rural survivors has been identified in a recent review [14]. A survey of rural survivors reported that only 38% and 10% were currently meeting aerobic and resistance training guidelines for survivors, respectively [12], demonstrating a clear need to increase accessible exercise programs for this population.

While current initiatives are designed to increase the accessibility of exercise programs for rural survivors of cancer [15,16], additional opportunities exist in the development of novel rural-focused program designs. The Exercise for Cancer to Enhance Living Well study in Canada [15] provides an exemplary model of using and improving clinical networks to increase awareness of and access to exercise oncology programs for rural survivors. The intervention itself, however, mirrors the available supervised programs by replacing in-person services with telehealth services, similar to other telehealth adaptations in the United States [17], which may not address all barriers experienced by survivors in rural areas. Beyond these examples, most other currently accessible exercise programs for rural survivors are phone-based walking programs [14]. Given that supervised exercise programs consistently demonstrate greater improvements in CRF compared to unsupervised programs [8], the efficacy of these current programs may be limited by the lack of consistent supervision and the recommended resistance exercise. Continued innovation in program designs specifically for the rural population is required to truly reduce geographic disparities targeting both improved accessibility and efficacy.

The purpose of this single-arm feasibility study was to assess a novel telehealth exercise program designed specifically for rural survivors of cancer with CRF, with an emphasis on replicating the CRF improvements seen in clinically supervised exercise programs. The program addresses known participation barriers for rural survivors and uses a validated reference chart of CRF improvement in a supervised program combined with regular symptom monitoring and symptom-triggered intervention. Data collection and outcome selection were designed to assess recommended objectives for feasibility studies [18] including recruitment, data collection, intervention acceptability and suitability, and a preliminary evaluation of participant responses. It was hypothesized that the program would demonstrate overall feasibility based on these objectives, providing support for a larger efficacy study.

## Methods

### Study and Program Design

**Figure 1:**
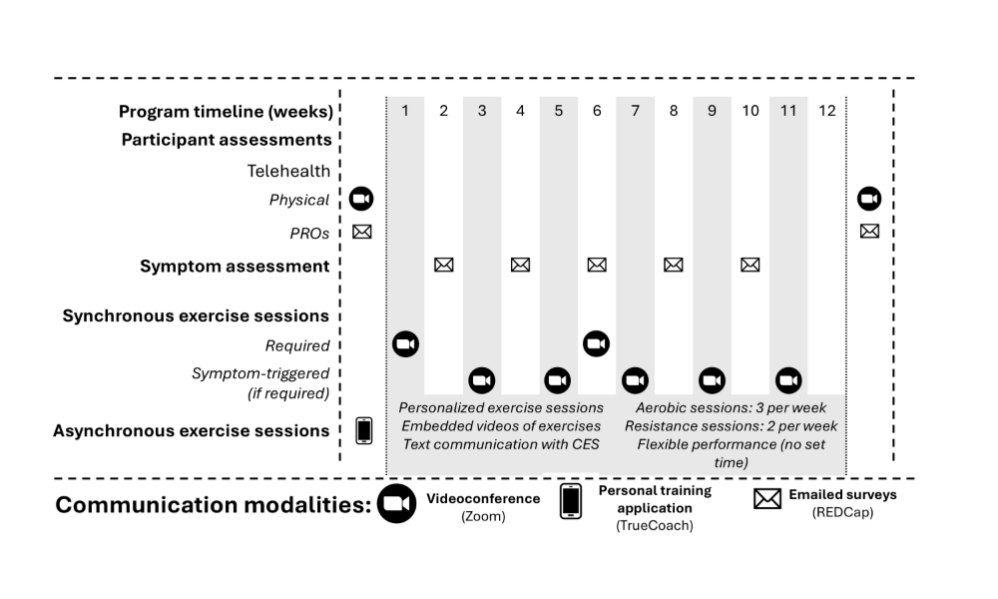
The design of the BfitBwell Telehealth Program with the timing of program components delivery. CES: cancer exercise specialist; PRO: patient-reported outcome; REDCap: Research Electronic Data Capture.

This was a single-arm clinical trial of the BfitBwell Telehealth Program (Figure 1), with assessments at baseline, program completion (12 weeks), and 6-month follow-up. Data collection occurred between November 2021 and September 2023. This 12-week telehealth exercise program was adapted from the clinically supervised BfitBwell Cancer Exercise Program [19,20]. Initial adaptations were made internally by program and research staff and designed to (1) address known barriers to exercise participation in rural survivors of cancer and (2) replicate the effects of a supervised clinical exercise program via telehealth. The specific barriers addressed were distance and transportation, lack of program flexibility (in regard to time and location), and lack of access to a knowledgeable exercise provider [12,13]. Participation in the BfitBwell Telehealth Program was decentralized, with centralized program oversight, to remove distance and transportation barriers. Three telehealth technologies were used: videoconferencing software (Zoom Video Communications, Inc), a personal training smartphone or internet app (TrueCoach Inc), and automated emails (REDCap [Research Electronic Data Capture]; Vanderbilt University [21,22]). The majority of exercise sessions were delivered asynchronously (ie, without real-time interaction between the participant and cancer exercise specialist [CES]) via the personal training app to provide program flexibility. Participants received scheduled (eg, recommended day of performance) and personalized exercise sessions via the app, but the timing of performance was determined by participants (eg, participants could complete sessions at any time of day, and “missed” sessions could be performed on later days). The personal training app also allowed embedded text communication between the prescribing CES and the participant following each individual exercise or exercise session. This within-app communication facilitated direct access to a knowledgeable exercise provider, as did regular engagement via automated emails for symptom monitoring.

Symptom monitoring was accomplished by plotting individual participant CRF scores on a validated reference chart of CRF improvements during a supervised exercise program [23]. The reference chart displays the typical course of CRF improvement for individuals in the supervised BfitBwell Cancer Exercise Program, against which the progress of individual participants can be easily monitored. The supervised BfitBwell Cancer Exercise Program has demonstrated effectiveness for improving CRF [19,20]. This type of reference chart has previously been proposed to inform personalized rehabilitation in other clinical populations [24,25]. In the BfitBwell Telehealth Program, CRF was monitored every 2 weeks and used to initiate symptom-triggered exercise sessions for participants failing to improve as predicted by the reference chart (see Symptom Tracking and Symptom-Triggered Sessions section). This served as the primary means of replicating the effects of a supervised program in a rural setting, as the reference chart allows individual participant progress to be compared to that of similar participants from a supervised program.

Finally, to promote participant safety in this remote telehealth context, a detailed safety and emergency response plan was developed. This plan included regular verification of the physical location of all videoconference sessions and the identification of a nearby “local support individual” (though this individual did not need to be present for assessments or sessions). Detailed plans of action were developed for emergent and nonemergent adverse events.

### Participants

Eligibility criteria for participation were adults ≥18 and ≤80 years of age, a diagnosis of any cancer type and any stage, completion of medical cancer treatment with curative intent within the past 12 months, or currently receiving treatment with no planned changes for the next 4 months. These criteria are similar to the patient population in the supervised BfitBwell Cancer Exercise Program and the associated CRF reference chart. Additional eligibility criteria included current moderate to severe CRF (>3 on a 10-point scale per National Comprehensive Cancer Network definition [26]), high-speed home internet and videoconference capable device (smartphone or laptop with camera), and residence in a rural area (defined here as >1-hour commute to a major city in Colorado or surrounding states with a known exercise oncology program, based upon review of a national program directory) [27]. Exclusion criteria included medical conditions that would impact the safety of, or participation in, a telehealth exercise program (eg, significant pulmonary or cardiovascular conditions and mobility-limiting orthopedic conditions). These conditions were self-reported on screening forms and individually reviewed by a licensed physical therapist (RJM) to determine eligibility. Safety was again assessed by the same therapist during the initial assessment (see Assessments section)).

A power analysis was not performed a priori, but a goal of 20 enrolled participants (with 15 completing the program) was set at study initiation. This recruitment goal was based primarily on the capacity of the supporting clinical exercise program combined with historical attrition rates of 25%.

### Recruitment and Enrollment

Initial recruitment efforts were made through clinical staff at a large urban cancer center that serves rural Colorado and surrounding states. Incoming and recent participants in the supervised BfitBwell Cancer Exercise Program were screened for eligibility. Recruitment efforts were later adapted to include targeted internet and social media advertisements (BuildClinical, LLC). Participants recruited through these efforts completed screening evaluations via emailed surveys or phone calls with study personnel. Eligible and interested participants were then invited to live videoconference sessions to further discuss the study and provide written informed consent. Enrolled participants were mailed all necessary equipment including a resistance exercise band set, a commercial fitness tracker (Withings Move, Withings Health), a smartphone tripod, an aerobic step (adjustable height 2-8 inches), and other necessary assessment equipment (eg, a measured length of rope, tape measure, and a pulse oximeter). Participants were incentivized to enroll by being allowed to keep all mailed study equipment, and gift cards were distributed to facilitate program and within-program survey completion.

### Primary Clinical Outcome

CRF was the primary clinical outcome and was assessed using the Functional Assessment of Chronic Illness Therapy—Fatigue Scale (FACIT-Fatigue) [28]. The FACIT-Fatigue is one of the most common measures of CRF with demonstrated reliability and validity in survivors of cancer [8,29]. It is a 13-item scale with scores ranging from 0 to 52, with higher scores indicating less CRF, asking participants to consider how they have felt during the past week. The minimal clinically important difference (MCID) of the FACIT-Fatigue scale has been identified as 3 points [30].

### Assessments

Initial and final assessments were performed via videoconference by a licensed physical therapist (RJM, separate from program CESs). In addition to collecting study outcomes, the initial assessment served as a final assessment of participant safety (based on the physical therapist’s clinical observations and judgment) and provided information used by the CES to personalize the exercise prescription. Demographic and cancer-related information were collected, and basic measures of physical fitness and function were performed similar to prior studies of telehealth assessments in survivors of cancer (adapted to be performed safely if the participant was alone) [31]. Physical assessment outcomes included single limb stance [32], gait speed [33], timed up and go [33], 30-second sit-to-stand [34], and a 3-minute step test (following the Tecumseh protocol [35]). Participants were interviewed about previous exercise experience, exercise preferences and goals, and available exercise resources (eg, home equipment and local gymnasiums). Patient-reported outcomes were then collected via emailed surveys. These included the FACIT-Fatigue [28], Functional Assessment of Cancer Treatment—General [36], Hospital Anxiety and Depression Scale [37], and the Godin Leisure-Time Exercise Questionnaire (GLTEQ, modified to estimate weekly moderate to vigorous physical activity and resistance exercise) [38]. Assessments occurred within the 2 weeks prior to intervention initiation and after completion. Patient-reported outcomes were again emailed to participants 6 months following program completion (follow-up assessment).

### Exercise Intervention

Exercise prescription followed current recommendations for survivors of cancer from the ACSM, specifically targeting 2 resistance exercise sessions and three 30-minute aerobic exercise sessions per week [9]. The prescribed exercise plan was delivered by 2 CESs employed by the supervised BfitBwell Cancer Exercise Program and resembled typical sessions for this program, previously described [19,20]. Both CESs (IAM and JJS) had an undergraduate degree in exercise science (or a related field), an exercise training certification (ACSM Certified Exercise Physiologist or equivalent), and at least 4 years of experience working exclusively with survivors in the supervised BfitBwell Cancer Exercise Program, and the primary CES (IAM) had an ACSM-CES certification. Resistance exercises targeted large upper and lower extremity muscle groups (using resistance bands, participant equipment, household objects, and body weight), and aerobic exercise was based on participant preference and available equipment (typically outdoor walking, treadmill walking, or stationary cycling). All exercise plans were personalized based on participant abilities, preferences, and available resources (established during the initial assessment).

### Exercise Sessions

One-hour, synchronous videoconference telehealth exercise sessions were scheduled with all participants in weeks 1 and 6. These sessions were performed in real time with the CES and participants interacting via live videoconference, mirroring an in-person supervised exercise session. Session content focused on providing education on exercise, demonstration and practice of proper exercise form, and supervised performance of exercises prescribed in the subsequent 6 weeks. All other exercise sessions (except the symptom-triggered sessions described in the next section) were delivered asynchronously via the personal training smartphone or internet app and included detailed individualized exercise content with embedded videos of a program CES performing the prescribed exercises. Participants had to indicate each exercise as completed within each session, creating a self-report measurement of asynchronous session completion. The embedded text communication between the prescribing CES and participant was regularly reviewed by the CES, who would then respond and adapt subsequent asynchronous exercise sessions, as necessary. The app was available via both smartphone app and internet browser.

### Symptom Tracking and Symptom-Triggered Sessions

Participants were emailed the FACIT-Fatigue 2 days prior to initiating the exercise program and every 2 weeks during the program, with daily reminders sent for up to 3 days. FACIT-Fatigue scores were monitored using the CRF reference chart to detect whether an individual’s progress matched the typical progress of similar individuals in a supervised exercise program, based on percentile rank established by initial scores. A symptom-triggered, synchronous videoconference exercise session was scheduled in the week following a FACIT-Fatigue score ≥3 points lower than the projected percentile at a given measurement time. The threshold was based on the MCID of the FACIT-Fatigue [30] in an attempt to ensure that symptom-triggered sessions were initiated due to a true lack of progress rather than normal variation or measurement error. Symptom-triggered session length ranged from 15 to 60 minutes, and a CES discussed program progress and challenges with the participant, with adaptations made to improve program response. These sessions were designed to mirror what would occur in a supervised program for participants who expressed a poor exercise response (eg, stated they continued to have fatigue) or in whom the CES identified a poor response (eg, performance plateau or decline). Common adaptations included changes in various exercise prescription components (Frequency, Intensity, Timing, and Type, via the ACSM guidelines [39]) and were made based on CES judgment in each occurrence. Emailed surveys also included a simple form for participants to ask exercise-related questions and request an additional videoconference exercise session the following week, even if not triggered by FACIT-Fatigue scores.

### Outcomes

#### Rationale

All outcomes were designed based on the recommended objectives, with guiding questions, for feasibility studies, as described by Orsmond and Cohn [18]. The current investigation focused on the use of collected objective data. While the ability of the research team to conduct the study and provide the intervention was not directly assessed, evaluation of other objectives allowed an indirect assessment (eg, successful data collection and attendance rates support the ability of the research team to perform these tasks). “Success” thresholds for feasibility outcomes were not set a priori, but rather outcomes were assessed holistically following the study as a means of assessing overall feasibility, providing the context of experience acquired while delivering the pilot program.

#### Recruitment

The number of participants screened, determined eligible, and ultimately enrolled were tracked, as was the recruitment rate (participants enrolled per month). The medium by which enrolled participants were recruited was recorded (eg, clinical referral or targeted internet and social media advertisements). The demographics of enrolled participants were summarized and separated by those who did and did not complete the program.

#### Data Collection

Completion rates of all clinical program outcomes from the initial, final, and follow-up assessments were calculated. Completion rates of the within-program emailed surveys (of 5 total) were calculated.

#### Intervention Acceptability and Suitability

Attendance rates were calculated for videoconference assessments and synchronous exercise sessions (including both the standard [2 sessions] and symptom-triggered sessions [up to 5 sessions]). Completion rates were calculated for asynchronous exercise sessions (based on downloaded session logs including previously described self-reported completion). Program safety was assessed by recording the number and nature of adverse events.

#### Preliminary Evaluation of Participant Responses

Participant responses to the intervention were evaluated by calculating changes in patient-reported and physical outcomes from initial to final assessments. Maintenance of participant responses following the intervention was investigated by calculating changes in patient-reported outcomes from final to follow-up assessments. FACIT-Fatigue change was also assessed on an individual participant level, with the number of participants achieving an MCID following the program determined. Within-program individual changes were visually investigated by plotting FACIT-Fatigue scores on the CRF reference chart, along with the occurrence of symptom-triggered sessions.

### Statistical Analysis

As a feasibility study with a small sample size, the majority of outcomes were summarized with descriptive statistics (counts, percentages, means, and SDs) and separately quantified for each assessment time point (initial, final, and follow-up) when appropriate. Following recommendations [18], the preliminary evaluation of participant responses was assessed using multiple approaches. In addition to the descriptive statistics provided for clinical outcomes and their change scores, the significance of change scores was assessed by statistically comparing these scores to 0 (ie, representing no change) using the nonparametric Wilcoxon signed rank test, as recommended for smaller studies as it does not assume a normal data distribution [40]. Significance was set at α<.05, established a priori, without correction for multiple tests, but with the presentation of individual *P* values facilitating further scrutiny. The clinical meaningfulness of changes in FACIT-Fatigue scores, the primary clinical outcome, was assessed by summarizing the participant-level outcomes and visual investigations (see Preliminary Evaluation of Participant Responses section). Only participants with available data were included in each analysis, with sample sizes reported for each analysis. All statistical analyses were performed using R statistical software (R Foundation for Statistical Computing).

### Ethical Considerations

This study was reviewed and approved by the Colorado Multiple Institutional Review Board (COMIRB # 20-2015) and registered on ClinicalTrials.gov (NCT04533165). All participants provided written informed consent prior to enrollment. Participants were incentivized to enroll by being allowed to keep all distributed assessment and exercise equipment (approximate US $200 value). Within-program survey completion was incentivized by providing a US $50 gift card if at least 3 were completed. Participation in the final assessment was incentivized with a US $50 gift card upon completion. Study data were stored securely in REDCap, and analyses used deidentified data.

## Results

### Recruitment

**Figure 2:**
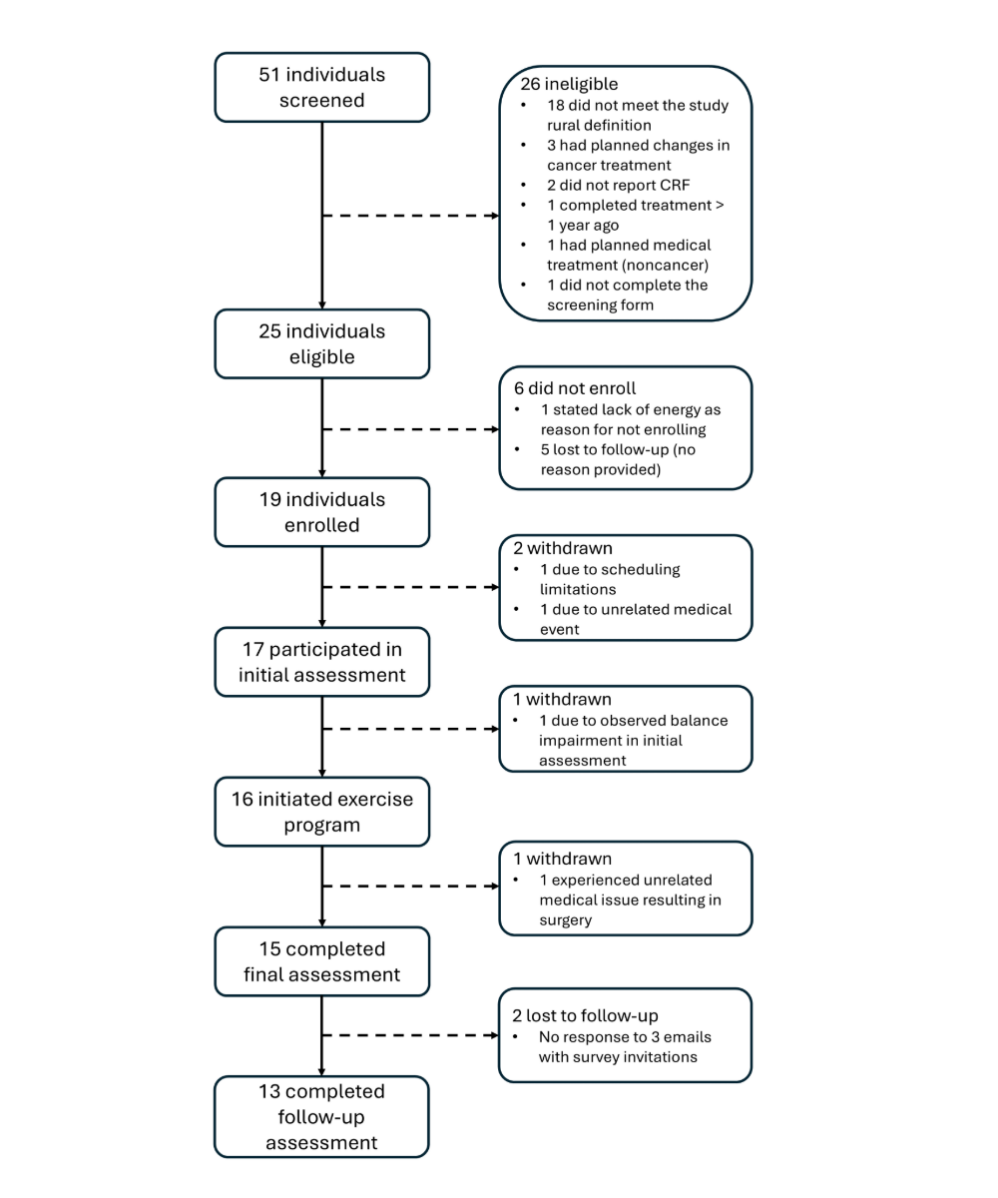
Illustration of screening, enrollment, and attrition during the study. CRF: cancer-related fatigue.

Figure 2 displays the flow of participants from screening to program completion, with attrition at each stage. Ultimately, 51 survivors of cancer were screened, 19 enrolled in the program, and 15 completed the program. All enrolled participants reported a “White” racial background. No participants reported Hispanic or Latino ethnicity. Of the enrolled participants, 14 (74%) were recruited through internet and social media advertisements, 4 (21%) were recruited from a registry of past participants in the supervised BfitBwell Cancer Exercise Program, and 1 person was recruited through clinical connections at a large urban cancer center. When all recruitment efforts were active, the maximum recruitment rate was 5 enrolled participants per month. Demographics and cancer-related data for enrolled participants, separated by those who did and did not complete the program, are shown in Table 1, and reasons for study withdrawal are shown in Figure 2 (note that most withdrawal reasons are unrelated to demographic and cancer-related data). All subsequent results include only participants who completed the program (and follow-up assessment when relevant).

One participant was withdrawn from the study due to the clinical determination that participation in a telehealth exercise program would not be safe due to previously unreported balance impairments, making the participant ineligible for the investigation. This safety determination was based primarily upon movement observation and objectively supported by poor performance on physical measures (single limb stance=3.5 seconds, gait speed=0.92 m/s, and inability to perform step test despite adaptation). Upon informing the participant of this decision, the study team facilitated a connection to a nearby facility providing supervised and skilled rehabilitation.

**Table 1:** Enrolled participant demographic and cancer-related information.

		Completed program (n=15)	Did not complete program (n=4)
**Age (years)**			
	Mean (SD)	60.7 (6.7)	55.5 (8.1)
	Range	49-71	44-63
**Sex distribution (female), n(%)**		11 (73)	4 (100)
**BMI (kg/m^2^)**			
	Mean (SD)	26.3 (4.1)	34.2 (11.9)
	Range	20.1-33.7	20.8-43.6
**Cancer diagnosis, n (%)**			
	Breast	6 (40)	3 (75)
	Colorectal	1 (7)	1 (25)
	Kidney	1 (7)	0 (0)
	Lung	1 (7)	0 (0)
	Multiple myeloma	2 (13)	0 (0)
	Non-Hodgkin lymphoma	1 (7)	0 (0)
	Ovarian	1 (7)	0 (0)
	Prostate	1 (7)	0 (0)
	Thyroid	1 (7)	0 (0)
**Receiving current treatment, n (%)**			
	Chemotherapy	3 (20)	0 (0)
	Immunotherapy	1 (7)	0 (0)
	Hormonal therapy	5 (33)	0 (0)
	None	6 (40)	4 (100)

### Data Collection

All physical assessment outcomes were successfully completed during the initial and final assessments except the 3-minute step test. In total, 3 participants were unable to maintain the required step rate for the duration of the test, 1 participant did not perform the test due to concerns of exceeding a safe heart rate (85% age-predicted maximum), and 2 participants experienced technical difficulties in measuring heart rate following completion of the test. All patient-reported outcomes were successfully completed at the initial assessment, and only 1 participant failed to complete both the Hospital Anxiety and Depression Scale and GLTEQ (presented last in emailed surveys) at the final assessment. In total, 13 (87% of those completing the program) participants completed all patient-reported outcomes at the follow-up assessment. The average completion rate of the within-program emailed surveys was 93% (70/75 surveys across all participants), with 12 (80% of those completing the program) completing 100%.

### Intervention Acceptability and Suitability

Attendance at initial and final videoconference assessments was 100% (30/30 possible assessments attended across all participants). Attendance at videoconference exercise sessions (weeks 1 and 6) was 97% (29/30 possible sessions attended), with only 1 participant missing 1 session. In total, 7 symptom-triggered sessions were initiated in 6 participants, with 6 (86%) of these sessions attended. A total of 3 participants requested 1 additional session, and 1 participant requested 2 additional sessions (non–CRF related). A total of 5 participants did not trigger or request any additional sessions. Participants received an average of 58 (SD 7) asynchronous sessions each, with an average of 49 (SD 11) indicated as complete (49/58, 84%, individual range 38%-100% [23/60 and 60/60 possible sessions completed, respectively]).

In total, 7 adverse events were reported in 6 participants. Of them, 2 were minor musculoskeletal issues (muscle strains) likely related to the exercise intervention. The remaining events were unrelated to the exercise intervention (minor musculoskeletal issues and illness).

### Preliminary Evaluation of Participant Responses

Table 2 displays the averages and changes in all outcomes at initial, final, and follow-up assessments. Group changes in all patient-reported outcomes from initial to final assessments were significantly different from 0 (in directions demonstrating improvement; all *P* values <.05, see Table 2 for individual values). Change in 30-second sit-to-stand was significantly greater than 0 (*P*=.005), and change in timed up and go trended toward being less than 0 (*P*=.09). At the follow-up assessment, only self-reported resistance exercise (GLTEQ-Resistance) significantly decreased from the final assessment (*P*=.01).

**Table 2:** Patient-reported and physical outcomes at initial, final, and 6-month follow-up assessments (n=15).

	Initial		Final					Follow-up			
	Values, n (%)	Mean (SD)	Values, n (%)	Mean (SD)	Change (SD)^a^	*P* value^b^	Values, n (%)		Mean (SD)	Change (SD)^a^	*P* value^b^
FACIT-Fatigue^c^	15 (100)	30.9 (7.4)	15 (100)	42.1 (8.0)	*11.2 (6.8)* ^d^	*.001*	13 (87)		40.9 (9.7)	–1.1 (3.4)	.29
FACT-G^e^	15 (100)	71.9 (11.6)	15 (100)	85.3 (13.3)	*13.4 (8.7)*	*.001*	13 (87)		85.4 (16.7)	0.8 (6.2)	>.99
HADS-A^f^	14 (93)	8.4 (5.7)	15 (100)	5.1 (4.3)	–*3.1 (3.6)*	*.002*	13 (87)		5.3 (4.1)	0 (2.2)	>.99
HADS-D^g^	14 (93)	6.2 (3.6)	15 (100)	3.8 (3.8)	–*2.2 (1.2)*	*.001*	13 (87)		4.1 (4.1)	0.1 (1.6)	.86
GLTEQ^h^-MVPA^i^ (minutes per week)	14 (93)	89.6 (105.4)	15 (100)	178.7 (171.1)	*95.4 (131.8)*	*.01*	13 (87)		148.1 (114.8)	–13.8 (129.0)	.66
GLTEQ-Resistance (minutes per week)	14 (93)	23.6 (37.3)	15 (100)	78.3 (32.1)	*50.7 (55.8)*	*.01*	13 (87)		21.9 (39.2)	–*51.2 (53.9)*	*.01*
Gait speed (m/s)	15 (100)	1.16 (0.15)	15 (100)	1.23 (0.10)	0.07 (0.17)	.12	—^j^		—	—	—
TUG^k^ (seconds)	15 (100)	9.2 (1.1)	15 (100)	8.8 (1.0)	–0.4 (0.9)	.08	—		—	—	—
30 s StS^l^ (repetitions)	15 (100)	11.7 (3.7)	15 (100)	13.3 (3.8)	*1.6 (1.7)*	*.005*	—		—	—	—
SLS-D^m^ (seconds)	15 (100)	28.2 (5.2)	15 (100)	26.4 (7.9)	–1.8 (4.8)	.10	—		—	—	—
SLS-ND^n^ (seconds)	15 (100)	29.0 (3.7)	15 (100)	28.2 (5.2)	–0.8 (6.7)	.79	—		—	—	—

^a^Change calculated from the previous assessment.

^b^Change statistically compared to 0 with the Wilcoxon rank sum test.

^c^FACIT-Fatigue: Functional Assessment of Chronic Illness Therapy—Fatigue Scale.

^d^Values in italics format emphasize *P*<.05, the a priori significance threshold.

^e^FACT-G: Functional Assessment of Cancer Treatment—General Scale.

^f^HADS-A: Hospital Anxiety and Depression Anxiety Scale—Anxiety Score.

^g^HADS-D: Hospital Anxiety and Depression Anxiety Scale—Depression Score.

^h^GLTEQ: Godin Leisure-Time Exercise Questionnaire.

^i^MVPA: moderate to vigorous physical activity.

^j^Not applicable.

^k^TUG: timed up and go.

^l^30 s StS: 30-second sit-to-stand.

^m^SLS-D: single leg stance—dominant (30 seconds maximum).

^n^SLS-ND: single leg stance—nondominant (30 seconds maximum).

**Figure 3:**
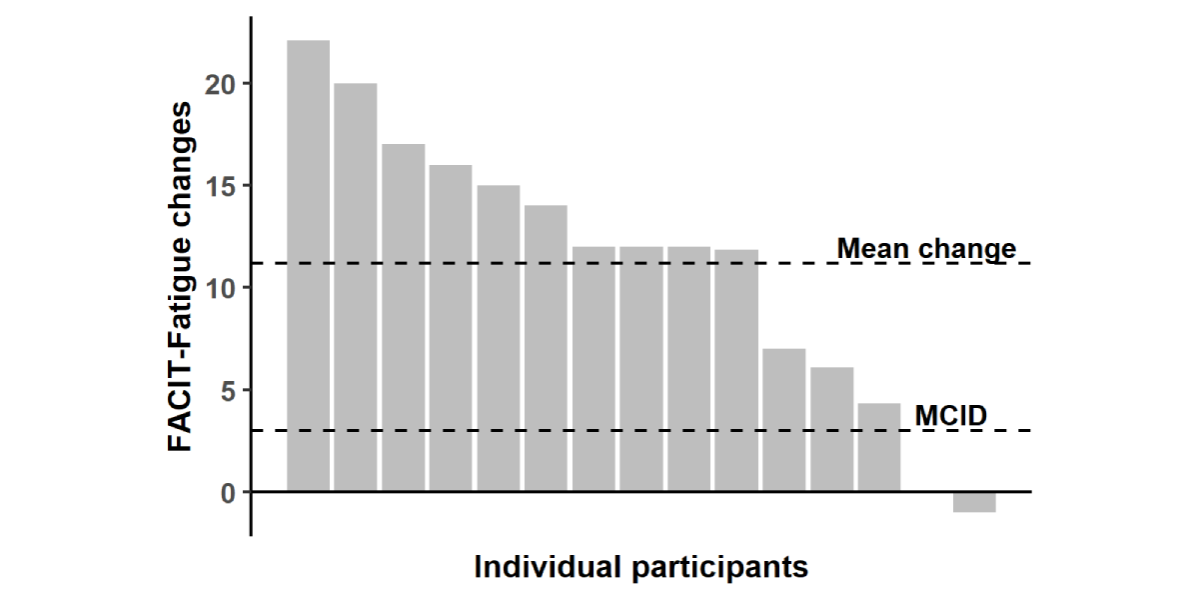
Waterfall plot displaying individual participant (n=15) changes in FACIT-Fatigue from initial to final assessments. The sample mean and FACIT-Fatigue MCID are displayed as dotted lines. Note that an increase in the FACIT-Fatigue score indicates improved fatigue. FACIT-Fatigue: Functional Assessment of Chronic Illness Therapy—Fatigue Scale; MCID: minimal clinically important difference.

**Figure 4:**
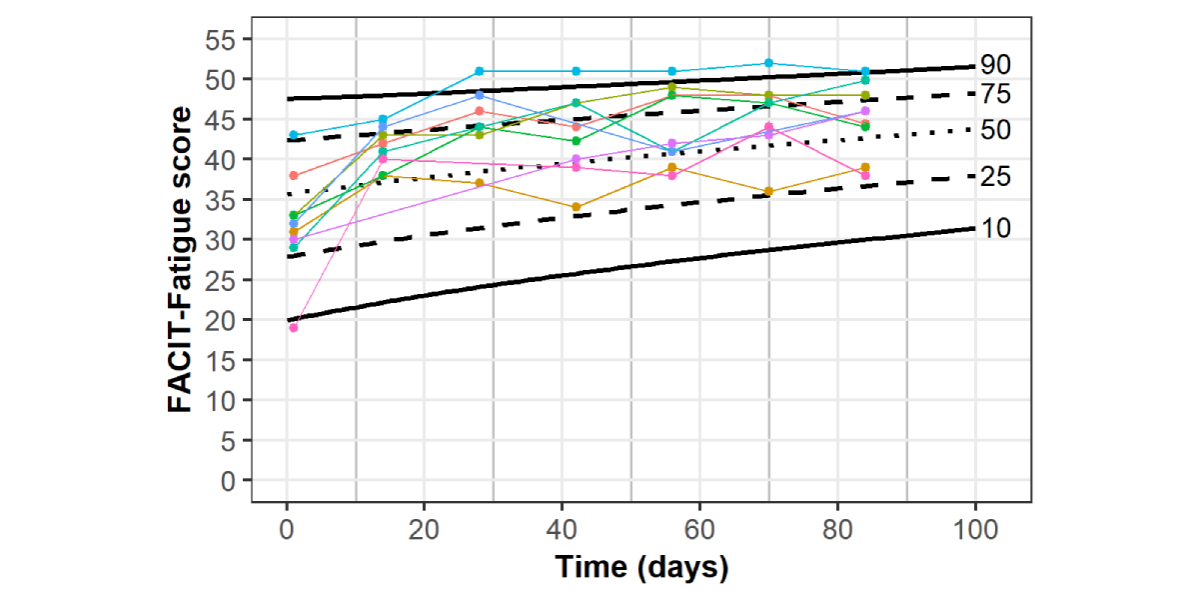
Within-program FACIT-Fatigue scores for participants with no required symptom-triggered exercise session (n=9) plotted on the cancer-related fatigue reference chart. FACIT-Fatigue: Functional Assessment of Chronic Illness Therapy—Fatigue Scale.

**Figure 5:**
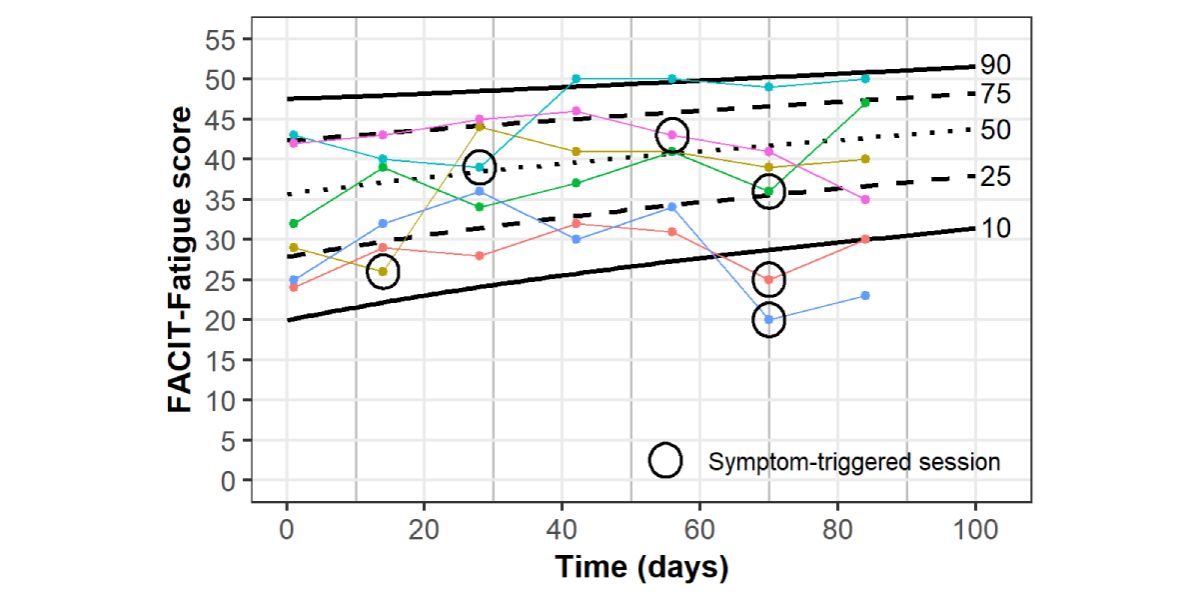
Within-program FACIT-Fatigue scores for participants requiring symptom-triggered exercise sessions (n=6) plotted on the cancer-related fatigue reference chart. Attended symptom-triggered sessions are indicated with circles. FACIT-Fatigue: Functional Assessment of Chronic Illness Therapy—Fatigue Scale.

Figure 3 displays the individual FACIT-Fatigue changes from the initial to final assessments. In total, 13 of 15 (87%) participants demonstrated an MCID in FACIT-Fatigue change. Figure 4 displays individual within-program FACIT-Fatigue scores, plotted on the CRF reference chart, for participants who did not require any symptom-triggered exercise sessions. Figure 5 displays individual within-program FACIT-Fatigue scores, plotted on the CRF reference chart, for participants requiring symptom-triggered exercise sessions, along with sessions attended. Of note, FACIT-Fatigue scores improved following symptom-triggered exercise sessions in 5 of 6 events, following previous declines.

## Discussion

### Principal Findings

This investigation assessed the feasibility of the novel BfitBwell Telehealth Program for rural survivors of cancer with CRF. Initial recruitment barriers were overcome through the adoption of alternative strategies (targeted internet advertisements). Outcome completion rates and intervention adherence rates were high, demonstrating the feasibility of data collection and intervention acceptability and suitability. Significant and meaningful improvements in CRF were observed at both the group and individual levels in the preliminary evaluation of participant responses. Based on these assessments of the recommended objectives [18], the BfitBwell Telehealth Program appears to be highly feasible, supporting the progression to a large efficacy trial.

### Comparisons to Prior Work

One of the main goals of this feasibility study was to adjudicate avenues for recruitment of eligible rural-living individuals. Given the barriers in accessing this population, it is not surprising that little work has been done specifically investigating the recruitment of rural-living people with cancer into exercise programs. A recent review of recruitment rates and strategies in exercise trials for survivors of cancer (not rural specific) revealed a low overall recruitment rate of 38%, with only 11% of included trials using web-based advertisements [41]. Additionally, “passive” strategies (including web-based advertisement) resulted in lower rates than “active” strategies (including clinic-based recruitment). Another larger exercise trial in rural survivors also demonstrated the success of active strategies such as community and clinical engagement [16]. Our findings contrast with these previous studies, in that only 5% of participants were enrolled as a result of active recruitment strategies (ie, clinical connections) at a large urban cancer center. A much more productive approach involved the use of targeted internet and social media advertisements. These approaches may indeed be considered more “active” than previous web-based approaches, as advertisements are routed to individuals based on their past internet activity. The reach of these advertisements to rural communities is undoubtedly broader than what can be achieved through clinic-based recruitment approaches. In our case, web-based recruitment might also provide a good match for individuals seeking a web- or telehealth-based program. More investigation is required into how to best engage and recruit rural survivors in exercise programming and associated clinical trials.

A unique aspect of our particular program was the reference chart–based monitoring of participant response to exercise along with the standardized addition of symptom-triggered sessions when participant progress deviated from the expected response. Lower than expected CRF improvements triggered additional sessions in 6 (40%) participants, and subsequent CRF scores appeared to improve following these symptom-triggered sessions (Figure 5). The purpose of these sessions was to mirror within-program exercise adaptations that are common in supervised clinical exercise programs in response to provider observations. While this facet of personalized exercise prescription is a relatively emergent feature in contemporary clinical research, the approach adheres to recommended best practices published by the National Comprehensive Cancer Network [42]. The current combination of symptom monitoring and symptom-triggered intervention is similar to a previous study of “chemotherapy-periodized” exercise, which demonstrated improved attendance when exercise prescription was adapted in anticipation of changing chemotherapy-associated symptoms during consecutive chemotherapy cycles [43]. Adaptations in the current program were made in accordance with the ACSM Frequency, Intensity, Time, and Type criteria of exercise prescription [39] and were based on CES expertise and information gathered during participant discussions.

Exercise attendance and program completion rates in our study were high. In total, 15 of 16 (94%) participants completed the program, and attendance and completion of all exercise sessions ranged from 84% (49/58 asynchronous sessions completed, on average) to 97% (29/30 videoconference sessions attended, across all participants). Additionally, within-program CRF monitoring was objectively successful, with an overall survey completion rate of 93% (70/75 surveys completed, across all participants). In total, 9 participants did not require symptom-triggered exercise sessions, completing the program largely autonomously and asynchronously. The lack of symptom-triggered sessions indicates similar CRF improvement compared to participants in a supervised exercise program, despite a reduced number of real-time (or synchronous) exercise sessions compared to many of these programs [19]. The asynchronous method of providing exercise programming and supervision used in this investigation may hold promise for improving both exercise efficacy and program efficiency in telehealth exercise programs.

The mechanisms underlying the observed CRF improvements remain uncertain, given the current investigation is a feasibility trial. Nonetheless, several strategies are likely to have contributed to engagement with the exercise prescription, facilitating exercise-associated CRF improvements in this cohort. The programmatic design specifically addressed three known barriers to exercise engagement in rural survivors of cancer, which are not frequently or consistently addressed in other programs (beyond reducing travel burden) [12,13]: (1) the need to travel was completely removed, (2) asynchronous programming provided flexibility for where and when exercise was performed, and (3) several methods of communication provided direct access to a knowledgeable exercise provider. For the involved CES, an unanticipated benefit of asynchronous programming was that the time commitment required may be lower than for a supervised program. While perhaps obvious, pragmatic and cost-effective strategies for improved exercise engagement may prove critical to overall program effectiveness, where personnel costs and reimbursement funds limit more intensive strategies. To this end, additional investigation of objective measures of program efficiency using asynchronous exercise programming is required.

### Strengths and Limitations

The primary limitation of this investigation is the lack of a control group and small sample size. Particularly in evaluating preliminary participant responses, the observed improvements during the program or in response to symptom-triggered sessions cannot be solely attributed to the intervention. However, several outcomes support the plausibility of the program influencing a meaningful improvement in CRF. First, the observed within-program improvements in CRF were similar to those documented in the supervised exercise program used to develop the CRF reference chart [23]. Second, significant improvements in CRF were observed immediately following the program, but no significant changes were observed in the 6 months following the program, indicating a lack of change due to time as well as the potential maintenance of program effects. Statistical analyses of pilot and feasibility trials, however, should be interpreted with caution [18]. Finally, on the participant level, the majority of participants (13/15, 87%) experienced a clinically meaningful improvement in CRF.

The sample in the current investigation is also likely biased in several ways. Given that participants responded to advertisements for an exercise intervention, the current sample is likely to reflect individuals who are able and willing to exercise. Additionally, the current sample is not large enough to adequately assess the contribution of various demographic and clinical variables on program adherence and response. The use of web-based recruitment methods and telehealth technology may discourage the participation of survivors without or not comfortable with technology. While the incorporation of this self-selecting sample population may contribute to a current “digital divide,” parallel efforts are actively reducing technological barriers through efforts to increase the availability and acceptance of adequate technology and internet connections. For example, the Broadband Equity, Access, and Deployment Program [44] seeks to expand high-speed internet access throughout the United States, facilitating a societal migration to technology-based health care interventions (including exercise). Given that the current investigation was performed during the COVID-19 pandemic, participants’ perspectives of and adherence to telehealth exercise may have been positively influenced in a manner unrepresentative of their more general context.

The used definition of rurality was developed independently by the research team, designed to target a population in need of services in the local region (Colorado and surrounding states). While this emphasis on local context is appropriate for this small feasibility study, future studies targeting larger populations in larger regions should use standardized rurality definitions to facilitate broader generalizations [10]. The telehealth tools used to facilitate access to this rural population may also contribute to a decrease in the fidelity of the prescribed exercise prescription. Specifically, only self-report completion data were available for asynchronous exercise sessions, limiting the knowledge of actual completion and performance. Future work could integrate additional technology (eg, improved activity trackers) to objectively assess the performance of these sessions.

Finally, while this investigation supports the feasibility of the BfitBwell Telehealth Program, it does not represent a comprehensive assessment of feasibility. While one set of recommended assessments was followed, multiple alternative definitions of feasibility and associated measures exist. Specifically, the assessment of intervention acceptability can be complex, including both quantitative and qualitative evaluations [45]. Nonetheless, the presented feasibility assessments provide strong support for further investigation of this program and its methods.

### Conclusions

The BfitBwell Telehealth Program used several telehealth modalities combined with regular within-program symptom monitoring and symptom-triggered intervention to deliver an exercise program to rural survivors of cancer with CRF. This investigation demonstrated high program feasibility, supported by positive assessments of recruitment, data collection, intervention acceptability and suitability, and preliminary evaluation of participant responses. Novel methods used by the program also provide a potential model for improving exercise program efficiency by using asynchronous exercise prescription. Future work should pursue large-scale efficacy testing, objective assessments of program efficiency, and systematic investigations of the effects of within-program exercise adaptations.

## Abbreviations

ACSM: American College of Sports Medicine
CES: cancer exercise specialist
CRF: cancer-related fatigue
FACIT-Fatigue: Functional Assessment of Chronic Illness Therapy—Fatigue Scale
GLTEQ: Godin Leisure-Time Exercise Questionnaire
MCID: minimal clinically important difference
REDCap: Research Electronic Data Capture

## Data Availability

The datasets generated and analyzed during this study are available from the corresponding author on reasonable request.
